# Mapping Semaphorins and Netrins in the Pathogenesis of Human Thoracic Aortic Aneurysms

**DOI:** 10.3390/ijms20092100

**Published:** 2019-04-28

**Authors:** Dornazsadat Alebrahim, Mangala Nayak, Alison Ward, Patricia Ursomanno, Rebecca Shams, Annanina Corsica, Rayan Sleiman, Kissinger Hyppolite Fils, Michele Silvestro, Ludovic Boytard, Tarik Hadi, Bruce Gelb, Bhama Ramkhelawon

**Affiliations:** 1 Division of Vascular Surgery, Department of Surgery, New York University Langone Health, New York, NY 10016, USA; Dornazsadat.Alebrahim@nyulangone.org (D.A.); Mangala.nayak@nyumc.org (M.N.); ras758@nyu.edu (R.S.); anna.corsica@nyumc.org (A.C.); Rayan.Sleiman@nyumc.org (R.S.); Kissinger.HyppoliteFils@nyulangone.org (K.H.F.); Michele.Silvestro@nyumc.org (M.S.); Ludovic.Boytard@nyumc.org (L.B.); Tarik.Hadi@nyumc.org (T.H.); 2 Department of Cardiothoracic Surgery, New York University Langone Health, New York, NY 10016, USA; alison.ward@nyumc.org (A.W.); pursomanno@gmail.com (P.U.); 3 Transplant Institute, New York University Langone Health, New York, NY 10016, USA; Bruce.Gelb@nyumc.org; 4 Department of Cell Biology, New York University Langone Health, New York, NY 10016, USA

**Keywords:** aneurysms, Semaphorins, netrins, extracellular matrix, vascular remodeling

## Abstract

Thoracic aortic aneurysm (TAA) is a complex life-threatening disease characterized by extensive extracellular matrix (ECM) fragmentation and persistent inflammation, culminating in a weakened aorta. Although evidence suggests defective canonical signaling pathways in TAA, the full spectrum of mechanisms contributing to TAA is poorly understood, therefore limiting the scope of drug-based treatment. Here, we used a sensitive RNA sequencing approach to profile the transcriptomic atlas of human TAA. Pathway analysis revealed upregulation of key matrix-degrading enzymes and inflammation coincident with the axonal guidance pathway. We uncovered their novel association with TAA and focused on the expression of Semaphorins and Netrins. Comprehensive analysis of this pathway showed that several members were differentially expressed in TAA compared to controls. Immunohistochemistry revealed that Semaphorin4D and its receptor PlexinB1, similar to Netrin-1 proteins were highly expressed in damaged areas of TAA tissues but faintly detected in the vessel wall of non-diseased sections. It should be considered that the current study is limited by its sample size and the use of internal thoracic artery as control for TAA for the sequencing dataset. Our data determines important neuronal regulators of vascular inflammatory events and suggest Netrins and Semaphorins as potential key contributors of ECM degradation in TAA.

## 1. Introduction

Thoracic aortic aneurysm (TAA) is a complex degenerative vascular disease distinguished by the progressive enlargement of the thoracic aortic vessel wall diameter, which can reach up to 1.5 times the normal aortic size [1]. With a three-year survival rate of 20% for patients with large dilated aneurysms, the disease is currently the 14th leading cause of death in the United States [2,3]. It is estimated that the incidence of TAA is ~5–10 in 100,000 people per year [4]. However, because TAA are asymptomatic, they can go undetected over a lifetime, making the actual incidence difficult to ascertain. In fact, the majority of TAA expand silently over time and are found incidentally on imaging. Increase in size of TAA is a predictor of devastating aortic dissection and rupture and is invariably fatal without prompt surgical intervention. 

The risk factors associated with TAA development are advanced age (>65 years), tobacco consumption, elevated blood pressure, atherosclerosis, family history, bicuspid aortic valve and genetic mutations such as in Marfan’s syndrome. Individuals with TAA are sometimes prescribed medications such as beta blockers and angiotensin II receptor inhibitors to reduce blood pressure to delay TAA growth and surgical intervention. However, there is no drug-based therapy targeted that prevents the life-threatening rupture associated with TAA, and surgical repairs remain the only treatment to alleviate the burden of patients. This is most likely due to the lack of insight regarding the mechanisms involved in the degeneration of the vessel wall in the pathophysiology of TAA. 

Over the past decade, cumulative efforts from several research groups have uncovered the deleterious roles of several matrix metalloproteinases (MMP) including MMP2, MMP9, MMP12 and MMP14 in promoting TAA by excessively degrading various components of the extracellular matrix (ECM), such as elastin fibers niched in the tunica intima and media. Notably, elastin fragmentation and thinned elastin lamellae are hallmarks of degenerative TAA. While the factors that contribute to the accumulation of matrix-degrading enzymes are not fully understood, some studies have suggested that the unique population of immune cells in aneurysmal tissue may contribute to this process. Evidence suggests that TAA aortic extracts collected from individuals suffering from Marfan’s syndrome, one of the heritable genetic manifestations of TAA characterized by mutations in the gene encoding fibrillin-1 (*FBN1*), are enriched with potent chemotactic factors that promote the increased migration of macrophages [5]. These data suggested that local alterations in *FBN1* gene could regulate the recruitment of macrophages, which could have a causative role in promoting pathological ECM remodeling in TAA. These results were recapitulated in murine models hypomorphic for fibrillin-1 (mgR) and demonstrated elevated macrophage migration when the cells were exposed to extracts of aortic lysates isolated from mgR mice [6]. Interestingly, Ramirez et al., who developed the mgR model, uncovered the persistent presence of macrophages both in the early and advanced aneurysms which clustered predominantly in the adventitial layer of the vessel wall [7]. They identified that fibrillin-1 was a potential substrate for the macrophage-specific MMP-12 that resulted in cleavage of the protein. Variants of fibrillin-1 have been shown to promote the activity of transforming growth factor-β (TGF-β) in the vascular wall. Based on clinical and experimental evidence, we hypothesized that the guidance signals could direct the accumulation of transmural macrophages and immune cells that manifest in TAA. 

Recent work from our group has established novel immunoregulatory roles for the neuronal guidance cues Netrin-1 and Semaphorins in guiding the immune response in the pathogenesis of cardiovascular insults such as atherosclerosis, obesity and abdominal aortic aneurysms [8,9,10]. Semaphorins and Netrins are widely recognized for their roles in axonal development and function by guiding the protrusion of axons to successfully establish communication networks [11,12]. Netrin-1 and Semaphorin3E (Sema3E) have previously been demonstrated to act as deleterious retention signals for lesional macrophages thereby promoting atherosclerosis [8,13]. Additionally, several research groups have established that B and T cells of the innate immune response express both Netrins and Semaphorins, although their precise role in regulating the immune response is still poorly understood. Here, using an unbiased RNA Sequencing transcriptomic approach, we identified increased axonal guidance pathway in the human specimens of TAA and further delineated the expression of some Semaphorins and Netrins stratified by TAA size. Our novel set of data have clinical relevance of our findings and could pave the way to detailed studies underlying the role of axonal molecules in the complex pathology of TAA. 

## 2. Results

### 2.1. Distinct Up-Regulation of Axonal Guidance Pathway in TAA 

**Figure 1:**
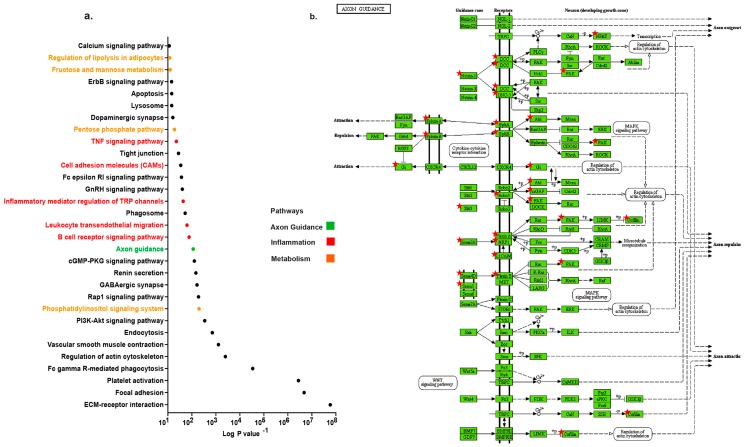
Distinct up-regulation of axonal guidance pathway. (**a**) Graphic representation of selected up-regulated pathways in thoracic aortic aneurysm samples compared to internal thoracic artery for the genes with *p* < 0.05 and fold change >2. (**b**) Schematic of KEGG enrichment pathway for the set of genes for axon guidance signaling in thoracic aortic aneurysm (TAA). The genes indicated by a star are predicted as increased in TAA.

TAA samples from 10 patients (Figure Sa) were compared to internal thoracic artery (ITA) samples from 3 controls. This generated a comprehensive set of ~60,000 genes that were detected. We acknowledge that some of the differential expression patterns might reflect differences coupled with intrinsic properties of the aortic tissues, it is however, unlikely that all of the significant changes detected are unassociated to TAA since ITA did not present any signs of pathology. In our analysis, we found 4831 genes that were significantly up-regulated and 2422 genes that were significantly down-regulated (*p* < 0.05) in TAA. Since pathological extracellular matrix remodeling by MMP have been described to promote TAA development, [14,15] we first screened for MMP variants in our dataset. Among the panel of MMP, Mmp12, 14, 24 and 28 mRNA were significantly increased in TAA compared to non-diseased control tissues (*p* < 0.05) (Figure Sb). In the quest to determine the significant pathways in the pathology of TAA we conducted pathway analysis of our data. Significantly up-regulated genes with fold change cut-off of equal to or greater than two were chosen for pathway analysis using David bioinformatic resources (version 6.8). The most up-regulated pathway was ECM receptor interaction, four metabolic-related pathways and five immune system networks sub-grouped into regulatory, signaling and migration categories (Figure 1a). Interestingly, the axon guidance pathway was significantly up-regulated (log_10_ *p* = 114, gene count = 19) in TAA. Members of the neuronal cue family linked to the guidance pathway are represented in the KEGG enrichment analysis diagram (Figure 1b). The expression of several members indicated by a star were significantly increased in TAA. All differentially expressed genes of axon guidance pathways are represented in Figure Sc. These data uncovered novel axonal pathway amplified in the development of symptomatic TAA.

### 2.2. Profiling Neuronal Guidance Subfamily Cues in TAA

**Figure 2:**
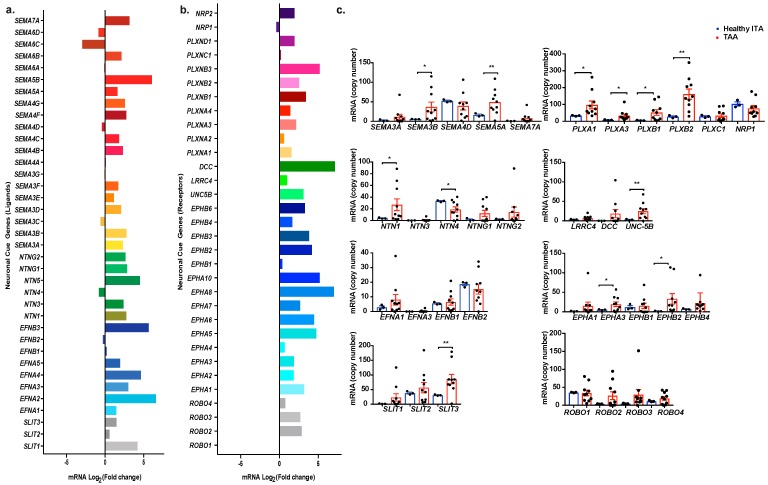
Profiling of neuronal guidance cues in TAA. (**a**) Transcript expression of neuronal cue genes (ligands) in thoracic aortic aneurysm compared to internal thoracic artery. Genes are clustered based on their family. (**b**) Transcript expression of neuronal cue genes (receptors) in thoracic aortic aneurysm compared to internal thoracic artery. Genes are clustered based on their family. (**c**) Profiling and analysis of mRNA copy numbers, identified by RNA-Seq, of the neuronal cue genes involved in axon guidance pathway. Genes are grouped by families of ligands and receptors. * *p* < 0.05, ** *p* < 0.01.

We further characterized individual genes of interest within the axonal guidance network. An overview of axonal guidance cue ligands and their receptors distribution and fold change expression classified by subfamily is represented in Figure 2a,b. A cursory look indicates an increased expression in most transcript levels in TAA. Bars indicate the relative increase or decrease of log_2_ fold changes of each gene. Increase in expression indicated by bars to the right and decreased expression bars are represented on the left. Apart from Sema6D, Sema6C, Sema4D, Sema3C, NTN-4, and EFNB2, the rest of the ligands had a higher relative expression in TAA tissue than healthy tissue (Figure 2a). Of all the neuronal cue receptor genes, only NRP1 and ROBO1 showed decreased expression in the TAA samples compared to internal thoracic artery samples. These results were further validated with the individual comparison of mRNA copy numbers of these genes and their respective receptors in healthy and TAA tissues. We grouped genes by their ligand and receptor families. Of the 34 genes that we explored further, 15 were significantly up-regulated in TAA tissue (*p* < 0.05) either the ligand or the corresponding receptor. The expression of NTN-4 and EFNB2 were decreased in TAA tissue as opposed to the remainder of the genes which increased in TAA. Semaphorins, Netrins and their receptors, including Plexins and Unc5b, were the most prominent families of genes significantly increased. Among these up-regulated neuronal guidance cues, we observed Sema3B (*p* = 0035), Sema5A (*p* = 0.0087), NTN-1 (*p* = 0.0467), and Plexin B1 (*p* = 0.0172) (Figure 2b). These results indicate an overall increased gene expression of neuronal guidance cues in TAA tissue, suggesting that neuronal guidance cues could contribute to the pathogenesis of TAA. 

### 2.3. Transcript Analysis and Protein Localization of Netrins and Semaphorins in TAA Tissue 

**Figure 3:**
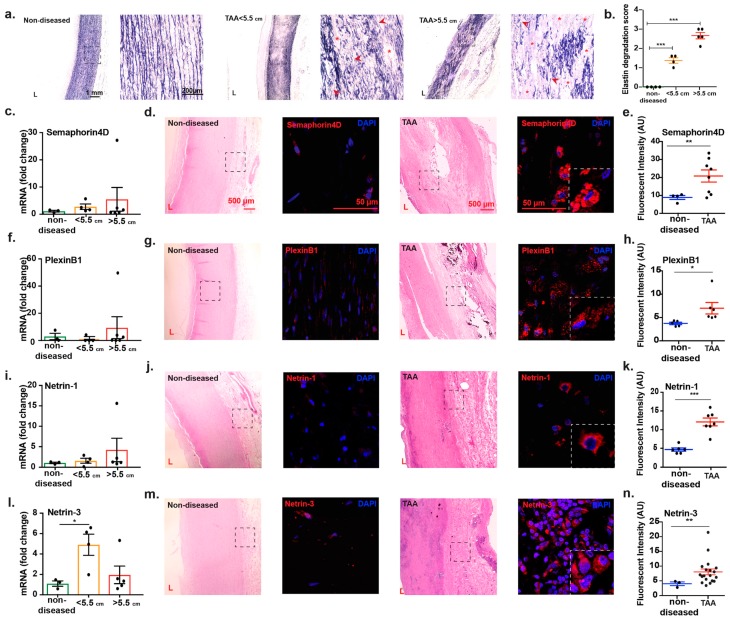
Transcript analysis and immunohistochemistry of Semaphorins and Netrins. (**a**,**b**) Elastin staining (Verhoeff-Van Gieson) and degradation score in non-diseased and TAA sections of size as indicated. Dashed boxes indicate magnified areas. Scale bars represent 1000 µm and 200 µm in low and high magnified images. Arrow indicate broken elastin fragments and stars indicate areas of complete elastin degradation. *** *p* < 0.001. Non-diseased (*n* = 4), TAA < 5.5 cm (*n* = 4), TAA > 5.5 cm (*n* = 5) (**c**) Quantitative PCR analysis of Semaphorin4D transcripts in non-diseased (*n* = 3), TAA < 5.5 cm (*n* = 4), TAA > 5.5 cm (*n* = 6). (**d**) Hematoxylin and Eosin (H & E) image and representative immunofluorescence staining of Semaphorin4D (red) and Dapi (blue) in non-diseased and TAA sections of human aorta. Dashed boxes indicate magnified areas of immunofluorescence staining (**e**) Quantification of Semaphorin4D immunofluorescence staining in non-diseased and TAA. (**f**) Quantitative PCR analysis of PlexinB1 transcripts in non-diseased (*n* = 3), TAA < 5.5 cm (*n* = 4), TAA > 5.5 cm (*n* = 6). (**g**) H & E image and representative fluorescence staining of PlexinB1 (red) and Dapi (blue) in non-diseased and TAA. (**h**) Quantification of PlexinB1 immunofluorescence staining in non-diseased and TAA. (**i**) Quantitative PCR analysis of Netrin-1 transcripts in non-diseased (*n* = 3), TAA < 5.5 cm (*n* = 4), TAA > 5.5 cm (*n* = 5). (**j**) H & E image and representative immunofluorescence staining of Netrin-1 (red) and Dapi (blue) in non-diseased and TAA sections of human aorta. Dashed boxes indicate magnified areas of immunofluorescence staining (**k**) Quantification of Netrin-1 immunofluorescence staining in non-diseased and TAA. (**l**) Quantitative PCR analysis of Netrin-3 transcripts in non-diseased (*n* = 3), TAA < 5.5 cm (*n* = 4), TAA > 5.5 cm (*n* = 5). (**m**) H & E image and representative immunofluorescence staining of Netrin-3 (red) and Dapi (blue) in non-diseased and TAA sections of human aorta. Dashed boxes indicate magnified areas of immunofluorescence staining. (**n**) Quantification of Netrin-3 immunofluorescence staining in non-diseased and TAA. * *p* < 0.05 ** *p* < 0.01 *** *p* <0.001. L = lumen. Scale bars represent 500 µm on H & E images. Magnified scale bars are 50 µm.

Since the size of aneurysms correlates with vessel wall damage, and therefore increases the risk of rupture, we performed Verhoeff-Van Gieson staining of the control and diseased tissues to detect elastin fiber alterations. As shown in the representative images in Figure 3a, we observed pronounced elastin damage characterized by fragmentation and degradation in TAA sections < 5.5 cm, this pattern was intensified in images captured from sections of TAA greater than 5.5 cm which revealed complete loss of elastin fibers in some sections of the aortic tissue. The quantification of elastin degradation revealed a significant difference between non-diseased and TAA < 5.5 cm and > 5.5cm (Figure 3b). These data suggested that increase in size of TAA correlates with advanced damage of the vessel wall consistent with previous studies. We therefore stratified the level of a selection of neuronal guidance cues based on the dilation of the aorta when surgical operation was conducted and the tissue collected. Interestingly, we found a positive association between the expression of Sema4D, Plexin B1, Netrin-1, and Netrin-3 genes and TAA size characterized by a trend in elevated mRNA in TAA diameters greater than 5.5 cm. However, our data did not reach statistical difference in our sample size (Figure 3c,f,i,l) probably due to differential complex translational and transcriptional regulatory mechanisms at stake within the TAA microenvironment or associated with technical postmortem tissue collection. We found that Netrin-3 mRNA expression was significantly increased in smaller-sized TAA compared to non-diseased control samples (*p* = 0.0313) (Figure 3l). However, the difference dropped when compared to larger TAA sizes. Immunofluorescence staining of TAA sections demonstrated strong presence of Semaphorin4D and its receptor Plexin B1 and two members of Netrin family, Netrin-1 and Netrin-3 protein in the adventitia and media of diseased tissue coincident with tissue damage. In comparison to the faint levels of neuronal guidance proteins that were detected in non-diseased, cadaver aortic sections (Figure 3d,g,j,m), quantification of immunofluorescence images showed significant increase in protein level of these neuronal cues and their receptors, which is consistent with the RNA sequencing transcriptomic results (Figure 3 e,h,k,n). Altogether, these findings point to instrumental yet unidentified roles for these neuronal guidance cues in TAA.

## 3. Discussion

In this study, we conducted a whole genome transcriptomic analysis of genes involved in the pathobiology of TAA. Through an unbiased screening approach, we identified several canonical networks such as calcium signaling and regulation of actin cytoskeletal signaling pathways, which have been previously described in TAA [1,16]. Notably, several members of the MMP family including 12 and 14 were significantly increased. This was associated with elevated TNFα signaling pathway. TNFα has previously been shown to induce the production of several MMP, including MMP14 [1,17,18]. Cross-talk between canonical mechanistic networks in TAA validates the robustness of our samples and screening method. Interestingly, pathway analysis uncovered novel axonal guidance genes that were distinctly elevated in TAA. In a recently published study, this pathway was also detected in a larger cohort of patients suffering from TAA although the authors did not focus their analysis on these axonal cues [19]. Notably, two of the most essential families of genes in the axon guidance pathway, Netrins and Semaphorins were increased in TAA aortic tissue suggesting they could have a causative role in the degenerative mechanisms in TAA development. Signaling cascades mediated by these cues could be integrated into other described defective routes, such as TGF-beta-regulated pathways. Alternatively, there is a growing number of studies demonstrating additional roles for Semaphorins and Netrins outside the central nervous system such as regulating the innate and adaptive immune responses and angiogenic processes that are associated with TAA. Therefore, these cues could also contribute to the pathogenesis of TAA via such mechanisms [13,20]. 

The role of neuronal guidance cues in the pathogenesis of the aneurysmal disease is burgeoning. In a pioneer study, we uncovered the deleterious role of macrophage-derived Netrin-1 in promoting the development of abdominal aortic aneurysms [8]. Single-Cell RNA sequencing of aneurysmal murine aortas revealed the enrichment of Netrin-1 in transmural macrophages and uncovered crosstalk mechanisms with adjacent vascular smooth muscle cells. Netrin-1 released by macrophages induced the sustained activity of MMP3 which induced elastin fragmentation. This was reversed in mice with conditional loss-of-function of Netrin-1 in macrophages. Although the etiology of TAA does not fully overlap with that of abdominal manifestations of the disease, here our data show that several members of the Netrin family including the ligands Netrin-1 and Netrin-3 and the receptor Unc5b were increased in TAA. The Netrin-1/Unc5b axis has been shown to play a role in the recruitment of circulating inflammatory cells in tissues. Our data suggest that signaling events through Netrin-1/Unc5b could guide the inflammatory response in a broader spectrum of degenerative vascular diseases analogous to abdominal aneurysms. As such, we show that multiple inflammatory and immune response related pathways, including cell adhesion molecules and the TNF signaling pathway were increased in TAA, invoking regulatory inflammatory mechanisms underlying the development of the disease. Future analysis that utilize murine models of TAA and loss-of-function of Netrin-1 would be useful to dissect the direct role of Netrin-1 in TAA.

Consistent with other studies, our data show that several members of the MMP family were increased in our cohort [21,22,23]. This was paralleled by heightened calcium signaling pathway and inflammatory mediator regulation of transient receptor potential (TRP) channels in TAA. Increased calcium signaling has previously been shown to correlate with increased aortic diameter [24,25]. Functional active sites of MMP require calcium ions to actively degrade the constituents of the extracellular matrix. Interestingly, the binding of Netrin-1 to its receptor Neogenin-1 has been shown to promote intracellular calcium influx in vascular smooth muscle cells and in neurons [8,26,27]. The size of TAA is one of the main indicators of life-threatening rupture and thoracic aorta diameter measurements of 5.5 cm is considered as a decisive indicator of surgical intervention [28]. Our results indicate an increased loss of extracellular matrix characterized by elastin staining in TAA and demonstrate that this was amplified in TAA samples exhibiting diameters greater 5.5 cm. Stratification of the expression of neuronal cues by size demonstrated that although most of the mRNA investigated were increased in TAA > 5.5 cm, Netrin-3 mRNA revealed a biphasic expression which peaked in samples of smaller TAA but not in those of bigger diameters. This data suggested that some of the neuronal cues might intervene during early phases of the disease development while others might be transcriptionally triggered at later stages after initial elastin loss is observed. Further investigation using experimental murine models of TAA is required to delve into the role of each neuronal cues at critical stages of TAA.

Semaphorins are a versatile group of phylogenetically conserved, secreted or membrane-bound proteins [29,30]. They were initially identified for their role in regulating the synaptic connection patterns [10]. In the past decades, extensive studies have uncovered their novel immune-responsive functions characterized by enhancing the activation of B cells through CD72 [31,32] and mediating angiogenesis by directing blood vessel sprouting from endothelial cells [33,34]. Kessler et al. described a new etiology for the development of TAA. They demonstrated that the formation of new leaky micro-vessels in the aortic wall could be causative in provoking remodeling and matrix degradation of the media. They demonstrated that this pathogenic neo-angiogenetic process was independent of the pro-angiogenic factor, vascular endothelial growth factor (VEGF) [35]. Sema5A similar to Sema3A [36,37] have been shown to play crucial roles in regulating endothelial cell migration and angiogenesis [38]. Mice with loss-of-function of Sema5A exhibit defective branching and development of cranial vessels [39]. These associative data could predict that observations made by Kessler et al. are regulated by these Semaphorin members in TAA, which warrant further experimental evidence. 

Pioneer studies led by Kumanogoh et al. discussed the immunoregulatory functions of Semaphorins and uncovered their roles in key stages of immune system response such as immune cell trafficking and dendritic cell transmigration across the lymphatics [40]. So far, Sema4D also known as cluster of differentiation (CD100) is the only member of this extensive family described to be highly expressed by lymphocytes. Through interaction with CD72 and PlexinB1, it has been shown to regulate B cell response by dissociating Scr homology phosphatase-1(SHP1) from CD72 [41]. Sema4D has been described to play a role in the disruption of cell-cell adhesive connections, a hallmark signal of ECM degradation that is crucial in the development of TAA [42]. Although the expression of Sema4D mRNA did not reach a statistical difference in our cohort analysis, there was a clear increasing trend of its expression in larger aneurysms. Immunofluorescence staining of TAA sections revealed significantly increased protein expression of Sema4D and its receptor plexinB1 suggesting a causative immunoregulatory role in TAA. As such, five inflammatory and immune system related pathways were increased in our pathway analysis including leukocyte trans-endothelial migration and B cell receptor signaling system. These findings further affirm the inflammatory nature of TAA and draw attention to the Sema4D/plexinB1 axis as potential underlying mechanisms. 

The function of extra-neuronal roles of Netrins and Semaphorins is poorly defined and warrants further investigation as to their contribution to the degenerative pathogenesis of TAA. We hope to further shed light on the mechanistic development of TAA and broaden the scope of knowledge in this field.

## 4. Materials and Methods

### 4.1. Human Samples

All studies were conducted in accordance with NYU Langone Medical Center Institutional Review Board policies. Informed consent was obtained from each patient. Samples of thoracic aortic aneurysm and internal thoracic artery from patients undergoing coronary artery bypass grafting (CABG) surgeries were gathered upon open repair procedures at NYU Langone Medical Center, New York, NY, USA. Non-aneury smal aortic tissue samples were collected from donors with no evidence of aortic diseases at autopsies provided by LiveOnNY organization, New York, NY, USA. Tissue samples were oriented, formalin fixed, paraffin embedded and sectioned or kept frozen at −80 °C for further analysis. Patient information was collected by a questionnaire or from the patient’s file at the hospital. From 16 TAA patients (11 men, 5 women) 2 patients had dissections. None of the patients had genetic collagen disorders. The demographic information of the Thoracic Aortic Aneurysms patients used in the study is provided in Figure Sa.

### 4.2. RNA Isolation

RNA extraction from TAA or healthy tissue was done by using RNEasy fibrous tissue kit according to the manufacturer’s instructions (74704, Qiagen, Hilden, Germany). Concentration and quality of RNA were verified by a NanoDrop One microvolume UV-vis spectrophotometer (ND-ONEW-W, Thermofisher Scientific, Wilmington, DE, USA).

### 4.3. RNA Sequencing

RNA was isolated from 10 human aortas and 3 internal thoracic artery samples then processed using Clontech Low Input Kit according to manufacturer’s instructions to prepare RNA-Seq libraries. RNA was purified using AMPure beads and quality was verified by Bioanalyzer (G2939BA, Agilent Technologies, Santa Clara, CA, USA). The samples were run on a HiSeq 2500 (Illumina, San Diego, CA, USA) as paired-end reads, 50 nucleotides in length. The read mapping was done against the hg19 human reference genome using Tophat 2.0.9. HTSeq 0.6.1 phyton framework and hg19 GTF gene annotation (UCSC database) were used to process BAM alignment files. To identify differentially expressed gene Bioconductor package DESeq2 (3.2) was used. In order to control the false discovery rate of the value results, they were adjusted by the Benjamin and Hochberg’s method. Genes that had adjusted *p* < 0.05 were considered to be differentially expressed. To discover the network of regulators and canonical pathways associated with transcriptomic data, significantly upregulated genes (with fold change >2) were analyzed using the Go DAVID open resource [43], and the Kegg pathway database [44,45,46]. 

### 4.4. Real Time and Quantitative PCR

cDNA was made by using the iScript cDNA Synthesis Kit (1708890, Bio-Rad, Hercules, CA, USA). Quantitative real-time PCR was performed in triplicates on a Quant Studio 3 Real-Time PCR System (Applied Biosystems, Wilmington, DE, USA) using KAPA SYBR FAST qPCR Kits (KK4602, KAPA Biosystems, Wilmington, MA, USA). Results that were calculated by the comparative cycle method (2^−ΔΔCt^) were then analyzed by fold change over the housekeeping gene.

Primer sequences used:

Human *GAPDH*: F GAAGGTGAAGGTCGGAGTC. R GAAGATGGTGATGGGATTTC.

Human Semaphorin4D: F TCCTGAAAGCCCGACTCATC. R AAGACATCCCGCAGCACATT.

Human Semaphorin5A: F GGAACCTGTGTTATAGCATGGC. R GCACTGAGTCGTACCCTGG.

Human PlexinB1: F TGCAGCATTACAAGGTCCCA. R TCCGCTCTCCAGGGACATAA.

Human PlexinB2: F AGCCTCTTCAAGGGCATCTG. R GCCACGAAAGACTTCTCCCC.

Human Neuropilin1: F GGCGCTTTTCGCAACGATAAA. R TCGCATTTTTCACTTGGGTGAT.

Human Netrin-1: F GCAAGCCCTTCCACTACGAC. R CGACAGTTGAGGCAGACACCT.

Human Netrin-3: F ACATGGAGCTGTACCGACTGT. R AGGGTCTCGATAGAAGCCCTC.

### 4.5. Histological Preparation and Immunohistochemistry

Samples were fixed in formalin and embedded in paraffin and sectioned (7 µm). Sections were rehydrated by 8 successive washes of xylene, xylene/ethanol (equal volume), 100% ethanol, 95% ethanol, 70% ethanol and water. The following buffer was used for antigen retrieval, 10 mM Tris, 1 mM EDTA, 0.05% Tween20, pH = 9. Following is the list of primary and secondary antibodies that were used: Mouse anti-human Semaphorin4D antibody (ab212275, Abcam, Cambridge, MA, USA), Rabbit anti-human Semaphorin4D antibody (bs-6965R, Bioss antibodies, Woburn, MA, USA), Rabbit anti-human PlexinB1 antibody (ab90087, Abcam), Rabbit anti-human Netrin-1 antibody (ab126729, Abcam), Rabbit anti-human Netrin-3 antibody (bs-11059R, Bioss antibodies, Woburn, MA, USA), Goat anti-rabbit antibody Alexa Fluor 568 (A11011, Invitrogen, Wilmington, DE, USA), Goat anti-mouse antibody Alexa Fluor 568 (S11004, Invitrogen), 4′,6-diamidino-2-phénylindole (Dapi, 1: 50,000 dilution; D1306, Invitrogen). Samples were counterstained with Dapi and mounted. For Hematoxylin-eosin (H & E) staining, the sections were incubated in eosin 515 LT (3801619, Leica Biosystems, Buffalo Grove, IL, USA) and in hematoxylin 560 (3801575, Leica Biosystems). Images were visualized using a Zeiss LSM 700 confocal microscope (Carl Zeiss, Stockholm, Sweden). We used identical acquisition parameters, level of contrast and brightness to image controls and TAA samples. Quantifications were performed on different section from different patients without any prior setting modifications. Magnification scale bars are shown in each image. Verhoeff Van Gieson Elastin stain set (25089-1, Astral Diagnostics, West Deptford, NJ, USA) was used according to manufacturer’s instructions, for staining elastin figments after 7 µm human sections were deparaffinized and rehydrated as aforementioned. Samples from TAA group demonstrate different levels of degradation so we used a semi-quantitative grading system for the elastin degradation scoring, wherein 0 presents no degradation and 3 represents severe degradation [47].

Each section’s score was determined by the mean score of at least 3 sites on the section. 

### 4.6. Statistical Analysis

Data are presented as mean ± SEM or SD where appropriate. The statistical difference between groups was analyzed by using GraphPad Prism 7.0 software and determined by one-way ANOVA followed by Dunnett or Tukey test for multiple comparisons among more than two independent groups. For comparison among two groups, one or two-tailed t-test was used. *p* < 0.05 were considered significant.

## 5. Conclusions

To our knowledge, this is the first study to profile the expression of neuronal guidance cues in complex thoracic aneurysmal disease. Unbiased RNA sequencing of human TAA revealed upregulation of the axonal pathways in TAA. These interesting findings provide *de novo* evidence to the complexity and diversity of their roles and highlight their potential pathological function in in TAA. Notably, we explore putative signaling cascades initiated by Semaphorins and Netrins that are associated with the immuno-degenerative process in TAA. The current study is limited by its sample size and the use of ITAs, instead of non-diseased aortic tissue, as control group which could potentially have refined the results obtained from RNA-Sequencing analysis. Further studies will be required to determine whether manipulating these axonal cues in individuals with TAA could help reduce the risk of rupture and improve the outcome of surgical interventions.

## 6. Limitations of the Study

For RNA Sequencing analysis, we used histologically non-diseased and non-atherosclerotic ITAs as controls because due to logistic reasons we did not yet have access to non-diseased cadaver aortic tissues. ITAs share similar features with aorta and have also been used in previous studies as control samples for ascending and descending TAA [19,48]. Notably, both ITA and TAA samples were processed and analyzed within same experimental settings to avoid methodological artefacts. We therefore believe that the data generated by RNA sequencing through this comparison warrants interest of novel signaling pathways enriched in TAA. Importantly, a selection of neuronal cues identified in the screen were validated in TAA sections compared to adequate non-diseased thoracic aortic tissues. We acknowledge that the number of samples used in the study is relatively small and larger prospective studies are required to dissect their role in TAA in order to demonstrate the clinical relevance of targeting neuronal cues in TAA. 

## Supplementary Materials

Supplementary materials can be found at https://www.mdpi.com/1422-0067/20/9/2100/s1.
