# Supplementary material for: Mapping Semaphorins and Netrins in the Pathogenesis of Human Thoracic Aortic Aneurysms
Source: Int J Mol Sci. 2019 Apr 28;20(9):2100. doi: 10.3390/ijms20092100 (PMC6539328; doi:10.3390/ijms20092100)
Supplement: Supplementary file 1 [file ijms-20-02100-s001.pdf]

## Supplementary Figure

a.

| | TAA |
|---|---|
| N | 16 |
| Age (mean) | 62.75 |
| Men (%) | 68.75 |
| Smoking (%) | 62.5 |
| Aortic Valve Disease (%) | 37.5 |
| Coronary Artery Disease (%) | 25 |
| HTN (%) | 12.5 |
| HLD (%) | 12.5 |

b.

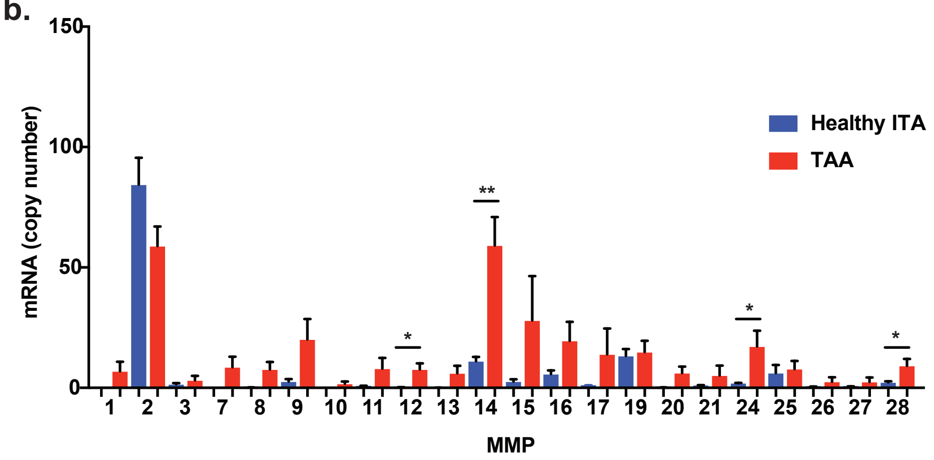


c.

| Upregulated | | | | | Downregulated | | | | |
|---|---|---|---|---|---|---|---|---|---|
| Symbol | ensembl_gene_id | FC | Log FC | P-value | Symbol | ensembl_gene_id | FC | Log FC | P-value |
| ABL1 | ENSG00000097007 | 1.420995146 | 0.506901627 | 0.021349351 | ARHGEF12 | ENSG00000196914 | 0.545327876 | -0.87480419 | 0.000391277 |
| EPHA3 | ENSG00000044524 | 3.664101609 | 1.873459511 | 0.042354919 | ABLIM1 | ENSG00000099204 | 0.253482862 | -1.980039887 | 4.83654E-05 |
| EPHB2 | ENSG00000133216 | 18.27305496 | 4.191645944 | 0.04385262 | ABLIM2 | ENSG00000163995 | 0.370227379 | -1.433516508 | 0.003228979 |
| GNAI2 | ENSG00000255749 | 1.665132471 | 0.735636956 | 0.005633343 | CFL2 | ENSG00000165410 | 0.065978165 | -3.921867545 | 0.020908462 |
| SRGAP1 | ENSG00000196935 | 4.412591747 | 2.141626275 | 0.018744029 | MAPK1 | ENSG00000100030 | 0.664208695 | -0.590291486 | 0.041708098 |
| SRGAP2 | ENSG00000266028 | 2.222493955 | 1.152179495 | 0.047768704 | NTN4 | ENSG00000074527 | 0.573107706 | -0.8031218 | 0.011317351 |
| CFL1 | ENSG00000172757 | 1.570435014 | 0.651164244 | 0.00992066 | NFATC3 | ENSG00000072736 | 0.578969266 | -0.788441329 | 0.020598241 |
| NTN1 | ENSG00000065320 | 13.99156426 | 3.80648536 | 0.015212636 | PPP3CB | ENSG00000107758 | 0.24991609 | -2.00048431 | 0.004393856 |
| NFATC4 | ENSG00000100968 | 4.206045215 | 2.072464359 | 0.021897796 | PPP3CC | ENSG00000120910 | 0.410045833 | -1.286142919 | 0.002635541 |
| PAK3 | ENSG00000077264 | 4.214620204 | 2.07540263 | 0.0140963 | PPP3R1 | ENSG00000221823 | 0.378627655 | -1.401148306 | 0.004024373 |
| PLXNA1 | ENSG00000114554 | 2.940834376 | 1.556225536 | 0.021623294 | SEMA6C | ENSG00000143434 | 0.128408795 | -2.961184073 | 0.003542479 |
| PLXNA3 | ENSG00000130827 | 4.484367392 | 2.164904479 | 0.029520759 | SEMA6D | ENSG00000137872 | 0.552002091 | -0.857254364 | 0.019451014 |
| PLXNB1 | ENSG00000164050 | 10.59986503 | 3.40597399 | 0.017199584 | | | | | |
| PLXNB2 | ENSG00000196576 | 5.808915495 | 2.538268843 | 0.001620802 | | | | | |
| SEMA3B | ENSG00000012171 | 6.827731148 | 2.771406251 | 0.034989912 | | | | | |
| SEMA4C | ENSG00000168758 | 3.501336567 | 1.807905748 | 0.038452894 | | | | | |
| SEMA5A | ENSG00000112902 | 3.051628132 | 1.609579167 | 0.008659819 | | | | | |
| SLIT3 | ENSG00000184347 | 2.776523707 | 1.473279714 | 0.00797024 | | | | | |
| SEMA4F | ENSG00000135622 | 6.723698318 | 2.749254994 | 0.03616964 | | | | | |
| UNC5B | ENSG00000107731 | 8.636344358 | 3.110420769 | 0.008724901 | | | | | |
| UNC5C | ENSG00000182168 | 3.920334803 | 1.970976868 | 0.015525507 | | | | | |

Supplementary figure legend:

(a) Demographic information of thoracic aortic aneurysm patients. (b) Analysis of MMP family mRNA copy number in thoracic aortic aneurysm (TAA) compared to internal thoracic artery (ITA) identified by RNA-seq. (TAA; n=10, ITA; n =3) *$P < 0.05$ **$P < 0.01$. The $P$ of the MMPs that are not mentioned are not significant. (c) Differentially regulated genes of axonal guidance pathway in thoracic aortic aneurysm samples.
